# Chronic oropharyngeal pain and medical nomadism in an Eagle’s syndrome patient: a case report

**DOI:** 10.1186/s13256-022-03372-0

**Published:** 2022-05-12

**Authors:** Yves Boucher, Agatha Mularski, Rufino Felizardo, Frédéric Tankere, Marc Dieb

**Affiliations:** 1 Laboratoire de Neurobiologie Orofaciale-LabNOF (EA7543), UFR. d’Odontologie, Université Paris Cité, 5 rue Garancière, 75006 Paris, France; 2 Service d’Odontologie, Hôpital Pitié-Salpêtrière, AP-HP, Paris, France; 3 Pole Odontologie, Hôpital Rothschild AP-HP Paris, UFR d’Odontologie, Université de Paris, Paris, France; 4 Sorbonne Université, CNRS, INSERM, ICM, centre MEG-EEG, AP-HP, HU Pitié-Salpêtrière, Service d’ORL et de chirurgie cervico-faciale, 75013 Paris, France

**Keywords:** Orofacial pain, Mechanical allodynia, Styloid process, Medical nomadism, Case report

## Abstract

**Background:**

Elongation of the styloid process associated with oropharyngeal pain and dysphagia is known as Eagle’s syndrome, a condition whose pathophysiology is still a matter of debate. Given its low prevalence and complex symptomatology, this syndrome is often misdiagnosed, leading to chronic pain and medical nomadism.

**Case summary:**

A 51-year-old woman of African origin with 3-year history of left-side oropharyngeal pain and worsening dysphagia consulted several health professionals. Medical and surgical treatments, including a sinus surgery and the extraction of three healthy teeth, did not improve her symptoms. Evaluation in an orofacial pain clinic revealed an asymmetrically elongated styloid process. Surgical shortening of the elongated styloid process provided complete pain relief and recovery of normal swallowing function.

**Conclusion:**

Based on this case report, the pathophysiology of Eagle’s syndrome is discussed, and the need for specific follow-up in a subpopulation of patients with asymptomatic styloid process elongation is highlighted.

## Key findings/Highlights

- Misdiagnosis of Eagle’s syndrome often leads to unnecessary and expansive medical nomadism.
- Eagle’s syndrome is a multifactorial syndrome.
- All physicians or clinicians (dentists, radiologists, etc.) should be sensitized and trained to detect and report styloid process elongation.

## Introduction

Eagle’s syndrome (ES) is a rare clinical entity that is associated with recurrent orofacial and cervical pain, being due to an elongated styloid process (SP) or a calcified stylohyoid ligament [1–4]. The syndrome was eponymized after the American otolaryngologist Watt W. Eagle, who characterized it in great detail in 1937 [5]. Findings consistent with ES, however, were already described in 1652 by the Italian surgeon Pietro Marchetti [1].

In the medical literature, ES is also referred to as stylohyoid complex syndrome, stylohyoid syndrome, styloid syndrome, elongated process syndrome, stylalgia, styloid–stylohyoid syndrome, styloid dysphagia, chronic styloid angina, temporal rheumatic styloiditis, stylocarotid syndrome, or Garel–Bernfeld syndrome (for a review, see [1]). Eagle subdivided this syndrome into two categories: classic type and carotid artery type. The classic type of ES is almost always observed after tonsillectomy, the most common presentation being a sensation of a foreign body in the oropharyngeal region, and dull and nagging pain without a lancinating (i.e., neuralgic) quality, during swallowing. This painful sensation may sometimes refer to the ear and the dentomaxillar region. The carotid artery type occurs after compression of the internal or external carotid artery by an elongated SP, but may also be present with other dysfunction and symptoms caused by irritation of the sympathetic nerve plexus. When the internal carotid artery is compressed, the pain is generally felt within the lateral region of the head or around the eye. In the case of external carotid artery compression, pain is referred to the infraorbital region and increases with rotation of the head. In addition, other symptoms such as transient visual loss, dizziness, or syncope may also occur. It should be noted that, despite their differences, both types of ES are thought to share a common etiopathogenic mechanism, namely local trauma resulting from excessive mechanical stimulation from an elongated SP. Nevertheless, it is not clear whether the resulting pain can be considered as inflammatory or neuropathic origin, the latter potentially resulting from chronic compression of the autonomic nerve branches surrounding the carotid artery in carotid artery-type ES or traumatic damage of primary sensory afferents. The prevalence of this syndrome appears to be very low, estimated at 0.16% [6], which together with its complex symptomatology, could lead to misdiagnosis. Here we report the case of a patient with Eagle’s syndrome, whose condition remained undiagnosed and unresponsive to therapy for over 3 years.

## Case report

A 51-year-old woman of African origin was referred to the Service d’Odontologie of the Hôpital Pitié-Salpêtrière (GHPS) in Paris, France, by her general practitioner. She described a 72-month history of pain in the left retroangulomandibular region, radiating to the ipsilateral ear and dentomaxillar region, of undetermined cause. The patient was referred to the specialized orofacial pain clinic of this service.

### Medical history

Anamnesis revealed that the pain began in 2013, when the patient started suffering from pain in the oropharyngeal region, radiating to the left maxilla and ear region, which was perceived as a foreign-body sensation, associated with painful chewing and swallowing, and noise in the ipsilateral ear. Acetaminophen (1000 mg, p.o., t.i.d.) provided only modest pain relief. After consulting a dentist in 2014, tooth #28 was extracted, without any improvement of her condition. Thereafter, she was referred to an otolaryngologist (ORL), who suspected sinusitis and treated it with antibiotics and corticosteroids, but no improvement was observed. The ORL then performed sinus meatotomy in 2015, but again no improvement of the symptoms was noticed. Finally, the patient consulted another dentist, who extracted tooth #25 and later tooth #38, after an episode of swelling of the left mandibular region unresponsive to antibiotic therapy (amoxicillin).

### Additional findings

The patient had undergone three C-sections and a hysterectomy. She also reported chronic stress attributed to a complicated marital life, which was not under medical treatment. In an episode of domestic violence in 2003, she was punched by her husband in the left retroangulomandibular region and behind the ascendant branch of the mandible (ramus). This was the only traumatic injury reported by the patient.

After this 3-year period of unsuccessful medical nomadism, in 2016 she consulted the orofacial pain clinic of the Hôpital Pitié-Salpêtrière in Paris, France. After a rigorous clinical and radiological examination by a senior expert practitioner (Y.B.), Eagle’s syndrome was diagnosed.

### Clinical findings

The patient reported a foreign-body sensation in the oropharyngeal region, and painful chewing and swallowing, mostly with solid foods, which had worsened over time. The pain was experienced almost every day, sometimes spontaneously, and it was enhanced by rotation of the head to the left. It could also be elicited by palpation of the tonsillar fossa. The pain was described as “aching during mouth opening” and “dull as a pharyngitis,” and was associated with a noise in the ispilateral ear. Pain quality was assessed by the DN4 questionnaire for neuropathic pain [7], which revealed only mechanical allodynia. The severity of the pain was rated 5/10 using the Numeric Pain Rating Scale (NPRS), and was partially relieved by acetaminophen (1000 mg, p.o., t.i.d.).

Orofacial evaluation according to DC recommendations [8] revealed a slight tumefaction at the left angle of the mandible without any modification in the appearance of the skin. Digital palpation revealed painful left masseter, mylohyoid muscle and the posterior part of the digastric muscles. Ear and nose examination was unremarkable. A left deviation of the mandibula during mouth opening was also observed. Intraoral examination revealed an asymmetry at the level of the palatine tonsils. The ipsilateral (left) tonsil seemed bent inside, and palpation of the oropharynx highlighted the left voluminous styloid process visible on orthopantomogram (see below).

### Radiographic findings

**Fig. 1:**
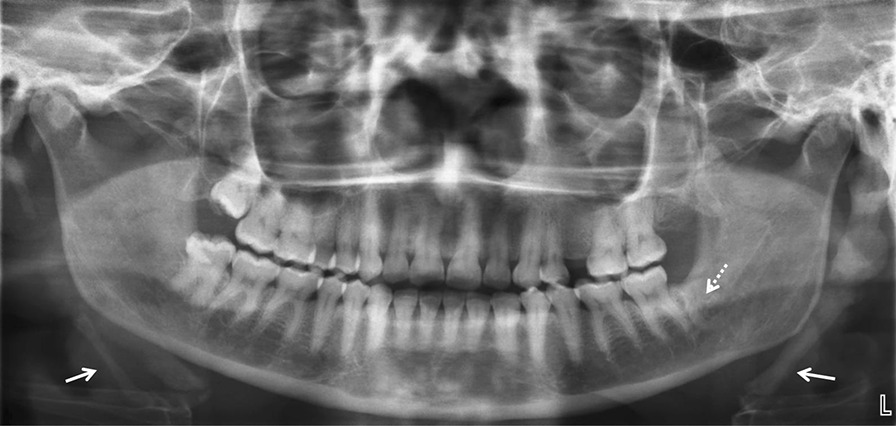
Orthopantomogram: teeth #25, #28, and #38 were extracted. Radiopacity (dotted arrow) is visible distal of #37, suggesting a residual root of #38 as a result of incomplete surgical treatment. Note that both styloid processes (solid arrows) are prolonged, but the left one was more prolonged and voluminous than the right

**Fig. 2:**
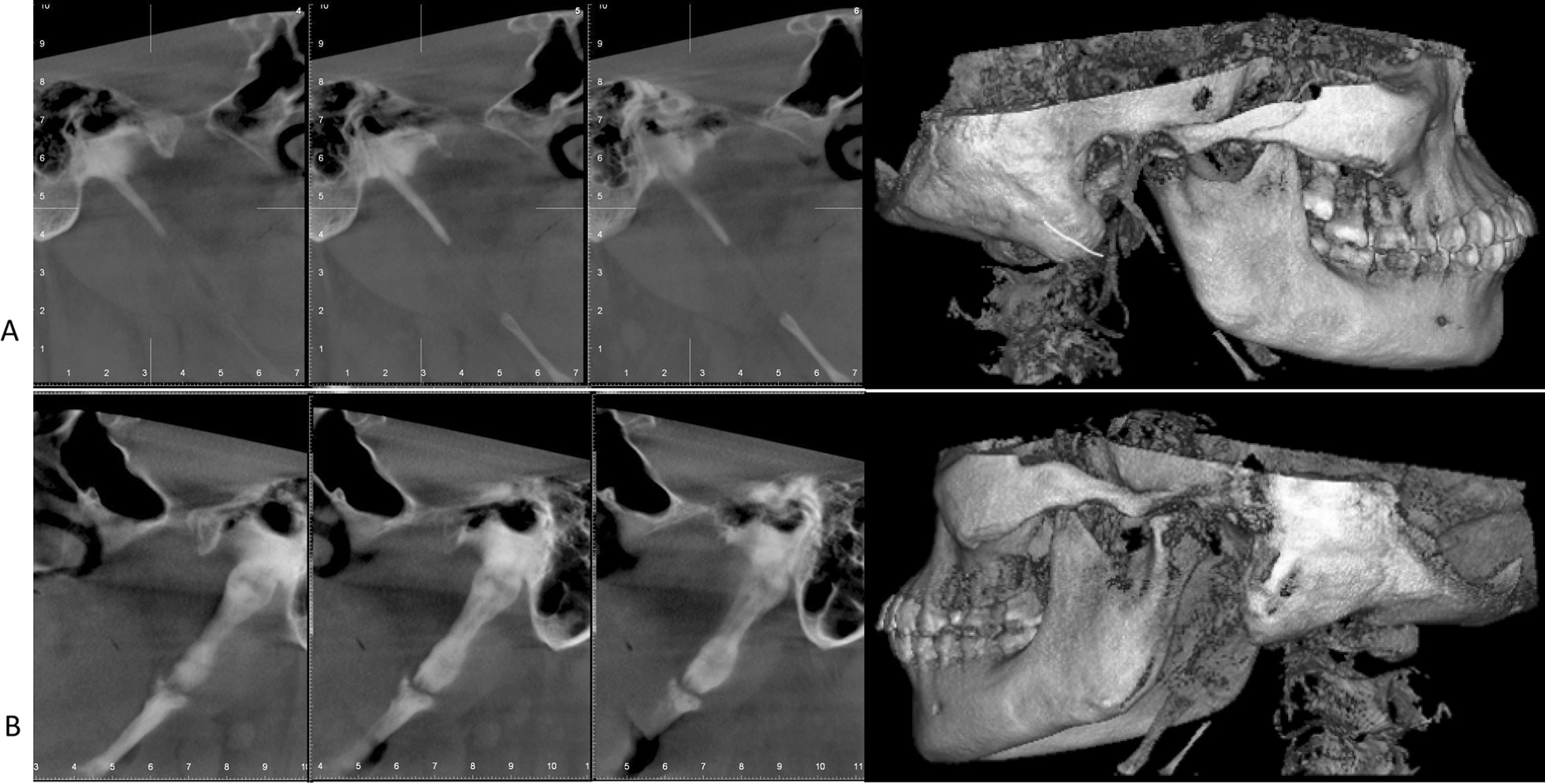
Dental cone beam computed tomography. **A** Right side: asymptomatic, **B** Left side, in which the styloid process corresponds to type II of Langlais’s classification; a superior mineralized segment is joined to an inferior mineralized one by a single pseudo-articulation that is located above the inferior border of the mandible

Visual inspection and panoramic radiography (Fig. 1) confirmed missing teeth #25, #28, and #38, and no dental/periodontal disease treatment. A radiopacity was visible distal of tooth #37, suggesting the presence of a residual root of #38 or an osseous condensation with no concomitant inflammatory process. As suspected by palpation, both SP were long, with the left one being more elongated. Cone beam computed tomography (CBCT) (Fig. 2) revealed a pseudo-articulation in the left SP: a superior mineralized segment joined to an inferior mineralized segment by a single pseudo-articulation with respective maximal dimensions of 37.3 × 13.1 mm and 32.5 ×10.5 mm, located above the inferior border of the mandible. This kind of SP corresponds to type II (pseudo-articulated) in Langlais's classification [9] (Table 1).

**Table 1:** Left: criteria for positive diagnosis of ES^20–22^; Right: differential diagnosis of ES^1^

Positive diagnosis	Differential diagnosis	
Medical history and clinical manifestations evocating one of two principal forms of ES: the classical or carotid form	Neuralgia	Glossopharyngeal neuralgia Superior laryngeal neuralgia Occipital neuralgia Sphenopalatine neuralgia Nervus intermedius neuralgia
A positive sign by digital palpation of the tonsillar fossa	Cephalalgia	Migraine Cervicogenic headache Carotidynia
Intratonsillar infiltration of local anesthesia (1 ml of 2% lidocaine) leads to complete pain relief	Oromandibular disorders	Temporomandibular joint disorders Unerupted or distorted third molar Faulty dental prostheses Sialolithiasis
Radiological checking by CBCT reveals SP elongation (> 2.5 cm) and/or its angulations	Ear–nose–throat diseases	Chronic tonsillitis Tonsillar calculi Otitis Mastoiditis Fracture of the hyoid bone Spasm of the pharyngeal Constrictor muscle Ernest syndrome Pterygoid hamulus bursitis Elongated pterygoid uncinus
	Others	Psychosomatic diseases Foreign bodies Inflammatory and neoplastic Processes in the oropharyngeal and esophageal areas Nuchal cellulitis and fibrositis Neck–tongue syndrome

Based on the medical history, clinical and radiographic findings, the final diagnosis of Eagle’s syndrome was made.

### Management of the Eagle’s syndrome

The patient was then referred to the ORL service of Hôpital Pitié-Salpêtrière, where a left stylectomy was decided, and performed using a classical external access technique, by an expert surgeon (F.T.) under general anesthesia [10]. At the 3-month follow-up visit, the pain was almost completely relieved. A slight limitation of mouth opening and mild pain sensation at left temporomandibular joint were still present, thus physical therapy was prescribed. At the second, third, and fourth recall visits at 2.5, 3, and 4 years after surgery respectively, total pain relief was observed and the functional discomfort had disappeared.

## Discussion

ES is a source of orofacial, oropharyngeal, and cervical pain that remains a diagnostic challenge for medical professionals. While being a rare medical entity, ES should be considered as a differential diagnosis in cases of oropharyngeal, cervical, and craniofacial pain of unknown origin. In the present case, the unnecessary removal of three teeth underlines the potentially severe consequences of its misdiagnosis on oral health.

The reported average “normal” length of the SP varies across the literature [11–13] because of the methodology employed [12]. An evaluation of the SP by three-dimensional (3D) computed tomography revealed that it can be classified into three groups according to its length: short (< 2 cm), long (2–4 cm), and elongated (> 4 cm) [14], the current reported case being in the latter category. However, It is important to notice that there is no relationship between the length of SP and the severity of pain [12, 15].

The etiopathogeny of ES is still a matter of debate. Eagle hypothesized that an iatrogenic procedure, such as tonsillectomy, leads to irritation of the stylohyoid ligament (SHL) and SP, which can induce inflammatory conditions such as osteitis, periostitis, or tendonitis, and subsequently reactive ossification of the SHL, resulting in SP elongation. However, only a minority of ES patients have a history of tonsillectomy [16, 17]. Furthermore, studies indicate a high prevalence of pediatric patients not being exposed to tonsillectomy who, nevertheless, showed definitive mineralization, in all or part, of the SHL [16]. Al-Khateeb *et al.* [15] suggested that tonsillectomy is not a major factor in the pathogenesis of the SP lengthening, and that chronic inflammation such as recurrent tonsillitis could probably play a more significant role in the development of ES. Supporting this view, the patient in our study had no history of tonsillectomy. We therefore hypothesize that the punch trauma could be the source of a chronic inflammation responsible of ES.

The diagnosis of ES is difficult for several reasons: firstly, as noted in the introduction, only ~4% of individuals having elongated SP develop ES. Thus, finding an elongated SP in a radiological examination is not sufficient to diagnose ES. Secondly, radiating and referred pain from the cervical region is a source of confusion for differential diagnosis. Moreover, as pointed out by Dieb *et al.* [18], it is difficult to state whether the resulting pain in ES has inflammatory or neuropathic origin, the latter potentially resulting from chronic compression of the autonomic nerve branches surrounding the carotid artery in carotid artery-type ES or neuropathic lesion of primary sensory afferents. In addition, the medical nomadism induced by an erroneous diagnostic can last for several years with some deleterious physical and social consequences [19]. Therefore, we suggest a revision of the definition of Eagle’s syndrome, as proposed by the International Classification of Headache Disorders 3rd edition (ICHD-3 beta version). This proposition aims to take into account the nature (inflammatory versus neuropathic) of this rare painful condition.

This case report highlights the medical nomadism the patient underwent after consulting several health professionals before receiving the correct diagnosis, following appropriate evaluation and differential diagnosis [1] (Table 1), and according to criteria proposed by several authors [20–22]. The management of ES is usually associated with antiinflammatory steroids as a first-line treatment [16], with surgical treatment usually proposed in cases of ineffective medical treatments [16]. Although ES is usually unilateral, most authors recommend a surgery during which both left and right SP are resected [10, 21, 22]. In our study, based on the radiological and clinical findings, the ORL surgeon shortened only the left SP, with a favorable outcome for both pain and function.

Finally, the erroneous diagnosis is probably due to a lack of knowledge of the clinical symptoms of ES and its differential diagnosis. A concise list of conditions should be considered for an accurate differential diagnosis of ES [1] (Table 1).

This case report might also provide insight into the pathophysiology of ES since, in the majority of ES patients, both SP are prolonged but the syndrome is usually observed unilaterally, as in the present case. Indeed, although both SP were elongated, pain was felt only on the left side, where the SP was longer. We hypothesize that an initial bilateral elongation of genetic origin [23] provided a risk factor for external trauma, thereby leading to an additional ossification in the left SHL and possibly the pseudo-articulation and increased volume of left SP (Table 2).

**Table 2:** Classification of styloid processes (SP)^9^

	Description
Type I	Elongated type The radiographic appearance of SP is characterized by an uninterrupted opacity
Type II	Pseudo-articulated type A superior mineralized segment is joined to an inferior mineralized segment by a single pseudo-articulation that is usually located above the inferior border of the mandible
Type III	Segmented type The SP is formed by more than two continuous osseous portions with areas of interruption either above or below the inferior border of the mandible

## Conclusion

While ES should be considered in the differential diagnosis of painful symptoms of the oropharyngeal region, it remains largely unknown by dentists. Misdiagnosis often leads to prolonged patient suffering, disability, and unnecessary medical procedures and expense.

The findings of this case report lead us also to consider ES as a multifactorial syndrome, thus we suggest that physicians should control and follow up every patient with asymptomatic SP elongation, especially when exposed to local chronic inflammation regardless of its origin: infection, surgery, external trauma, etc. Moreover, dental radiologists should report the presence of SP elongation even if the patient does not exhibit pain symptoms.

## Data Availability

Not applicable. According to the patient recommendation and the directives of the National Personal Data Protection Committee (CNIL) in France, patient data are authorized for use by our research team but not to be shared.
